# Neither responsive, nor responsible? Citizens’ understandings of political actors’ responsiveness and responsibility in the socio-economic governance of the EU

**DOI:** 10.1080/13501763.2023.2288236

**Published:** 2023-12-23

**Authors:** Claire Dupuy, Virginie Van Ingelgom

**Affiliations:** a ISPOLE, University of Louvain, Louvain-la-Neuve, Belgium; b F.R.S.-FNRS, Brussels, Belgium

**Keywords:** Responsiveness, citizens, agency, political actors, qualitative secondary analysis, abductive coding

## Abstract

The implications of the dilemma attributed to Mair between the responsiveness of political actors and their responsibility is considered from the perspective of citizens. The article analyses citizens’ understandings of political actors’ responsiveness and responsibility in their discourses on the SEGEU. We provide an in-depth abductive secondary analysis of three qualitative datasets collected in Belgium and France between 2005 and 2019. Most participants frame responsibility and responsiveness in terms that are close to Mair’s definitions. However, some challenge the principle of responsibility and elaborate an alternative understanding directed towards achieving the common good. Nevertheless, as participants agree that political actors are not responsive to them, but rather to market actors, there is no evident dilemma. Our analysis shows that political actors’ capacity for agency and choice, frames participants’ understandings of responsiveness and responsibility. This result becomes the basis for a reconceptualisation of the notion of responsiveness and representation from a citizen perspective.

## Introduction

Drawing from Mair’s analysis of member states’ responses to the Euro crisis (2013), the socio-economic governance of the EU (SEGEU) has been argued to feature a widening gap between national political leaders’ responsiveness towards their electorates, and their responsibilities to economic and political integration. As responsible decisions have prevailed over responsive choices in reforms of the SEGEU (e.g., Laffan, 2014; Mérand, 2021), as well as in domestic policymaking (e.g., Schäfer & Streeck, 2013), the classic debate about how governing actors ought to combine the tasks of political representation with good government has turned into an increasingly acute dilemma. Recently, however, analyses of decision-making around Next Generation EU suggest that Mair’s dilemma may be historically contingent rather than a defining feature of contemporary European politics. Not only have concerns about citizens gained more leverage at the EU-level (Mérand, 2021), but insights also suggest that these concerns have been included in the definition of responsible governmental decisions – which is referred to as ‘responsive responsibility’ (Crespy *et al*., 2024). This has reinvigorated discussion around what responsibility and responsiveness potentially entails to different groups of national and European actors in the context of the SEGEU.

This article considers citizens’ understandings of political actors’ responsibility and responsiveness in relation to the SEGEU, adding three main contributions to Mair’s analysis which centred on parties and governments (2014). Empirically, we integrate crucial actors, or specific subsets of them, who are suggested to be end-beneficiaries of responsive decisions. Relatedly, studying citizens’ understandings of responsibility and responsiveness prior to Next Generation EU is instrumental to illuminating the changes in the SEGEU, and the possible turn to ‘responsive responsibility’. Analytically, from a constructivist standpoint, the dilemma between responsibility and responsiveness is grounded in actors’ perceptions, only from which a dilemma may arise, become closed or be reframed. Consequently, we are equally attentive to citizens’ understandings. Finally, normatively, the implications of the dilemma, which for Mair poses potentially serious threats to the long-term legitimacy of national democracies and the EU polity (2008), rests on whether European citizens’ perceptions match Mair’s and others’ scholarly analyses. If citizens do not perceive a dilemma, does it matter if scholars do?

Citizens are virtually absent from the scholarship on the responsibility-responsiveness nexus which overwhelmingly focuses on governmental and party-political actors (but see Linde & Peters, 2020). To address this, we draw on the qualitative turn in EU studies, that examines citizen discourses surrounding European questions in their own terms, and ask: *how do citizens express understanding of political actors’ responsiveness and responsibility in their discourses on the socio-economic governance of the EU prior to the adoption of Next Generation EU?* First, we analyse what citizens discuss and how contentious it is, to reveal possible shared norms amongst citizens about the responsibility and responsiveness of political actors in the SEGEU. Secondly, we examine how citizens discuss responsiveness and responsibility, to analyse the cognitive frames they draw from when discussing these topics. The article thus contributes to a differentiated analysis of citizens’ reactions to European integration, which is attuned to its multiple dimensions, and goes beyond the average support or opposition to the EU (Duchesne *et al.*, 2013; Hobolt & De Vries, 2016).

Our study rests on a comparative and longitudinal design, devised to probe the existence of shared understandings across national cases, time and interviewees’ socio-economic background – which is our main focus, rather than a study of their variation. We conducted a qualitative secondary analysis of interview data from three primary datasets of focus groups generated in 2005–2006, 2013–2014 and 2019 covering Belgium and France (Beaudonnet *et al.*, 2022; Duchesne *et al.*, 2013; Mercenier, 2019). Belgium and France exhibit theoretically meaningful variation regarding citizens’ support for the EU, and political elites’ sometimes distinct strategies when it comes to endorsing the rules and principles of the SEGEU. We focus on the SEGEU as a means to distil how citizens understand political actors’ responsibility and responsiveness, and the possible dilemma between the two. Our analysis is abductive (Tavory & Timmermans, 2014; Vila-Henninger *et al.*, 2022); it is oriented towards theory-building by combining deduction and induction. We start from existing theories of how citizens understand political actors’ responsiveness and responsibility, paying particular attention to empirical instances that deviate from them. The analysis of these, alongside the broader corpus, grounds our theoretical efforts at generating a better fit between theory and data; and results in expanding our theoretical breadth as new concepts are brought into the analysis (agency and choice).

Empirically, our research shows that whilst citizens do discuss political actors’ responsiveness and responsibility in the context of the SEGEU, they do so in different ways. Political actors’ lack of responsiveness to ‘citizens’ is a norm shared amongst interviewees regardless of country, socio-economic background and point in time. Participants employ a cognitive framework that isolates agency and choice when observing political actors’ responsiveness to market actors; a response consequent upon deliberate decisions which left them with little choice. There is no shared norm amongst participants in the case of political actors’ responsibility as some of them question its very principle. The dominant understanding is to regard it as a set of institutional constraints stemming from the EU, whilst a few others argue for an alternative definition centred on achieving the common good. Despite this, participants share the same cognitive frame; namely political actors’ agency and choice, while disagreeing over whether compliance with institutional rules was a deliberate choice, or a justified necessity for political actors and whether the latter have any alternative left. Finally, the data shows few instances of Mair’s dilemma, as political actors’ responsiveness towards citizens is, in their minds, unquestionably non-existent.

Building on these findings, we theorize first, that citizens’ understandings of responsiveness are shaped by a trustee-type of relationship, whereby citizens trust that politicians will act in the common good, but do not necessarily expect them to fulfil electoral promises or react to shifts in public opinion. Secondly, consistent with Hay’s conceptualisation of the political (2007, 2014), we acknowledge the prevalence of political actors’ agency and choice in citizens’ understandings of responsiveness and responsibility.

In the remainder of the article, section 2 presents the state of the art. In section 3, we discuss our data and methods of data analysis. Section 4 focuses on our empirical analysis and section 5 presents our discussion and reconceptualisation of the notion of responsiveness and representation from a citizen perspective.

## State of the art: Citizens’ understandings of political actors’ responsibility and responsiveness

### State of the art

During the Euro crisis and its aftermath, the emphasis on fiscal discipline steered national governments economic policymaking, and also ensured that responsibility underlined reforms of the SEGEU. While empirical studies support Mair’s analysis of the growing dilemma domestic governments have faced between responsibility and responsiveness (Damhuis & Karremans, 2017; Karremans, 2017, 2021; Karremans & Damhuis, 2020; Karremans & Lefkofridi, 2020; Laffan, 2014), their results beg serious questions: have citizens made the same observations? Have they perceived the evolution of the trade-off(s) between responsible and responsive governments in the same terms as the scholarship suggests? Despite having considerable implications for the relationship between political actors and citizens (Mair, 2008), the body of work concerned with the responsibility-responsiveness nexus has remained largely oblivious to citizens’ perspectives (for an exception see Linde & Peters, 2020). This is problematic as, according to Mair himself, the growing gap between responsiveness and responsibility – or between what citizens might like political actors to do and what they are obliged to do – lies at the heart of the disaffection and malaise that suffuses democracy (Mair, 2014). Other scholarships, however, lend indirect empirical support to Mair’s analyses from a citizen perspective.

First, a burgeoning literature suggests that governments’ loss of autonomy in the context of international economic integration impairs political representation (Hellwig, 2020), and results in parties becoming increasingly aligned with external actors’ preferences (Ezrow & Hellwig, 2014). In this respect, economic integration weakens the capacity and/or willingness of elected politicians to be responsive to their constituents’ expressed preferences, which in turn leads citizens to believe that their concerns are ignored in policymaking (Holmberg, 2020).

Secondly, an additional strand of research suggests that the political constraints imposed by economic integration decrease electoral turnout because the perceived benefits derived from voting are lowered (Steiner, 2016; but see Vowles, 2008). Whilst this negative relationship between international integration and electoral participation has been empirically confirmed at the aggregate level (e.g., Steiner, 2010), it is less apparent at the individual level (but see Le Gall, 2018; Steiner, 2016). This finding is indeed still contentious. The association between external constraints, governments’ political efficacy and turnout has been accounted for in the literature on the debt crisis (Haüsermann *et al.*, 2018; Ruiz-Rufino & Alonso, 2017); whereas Kostelka and Blais (2021) recently found that economic globalisation does not decrease turnout in the long run.

Thirdly, studies demonstrate the centrality of voters’ attribution of responsibility to the EU (Hobolt & Tilley, 2014). They show that perceptions of EU responsibility are likely to moderate government accountability at national (Costa Lobo & Lewis-Beck, 2012) or European level (Magni-Berton *et al.*, 2021). Overall, research reports that either the existence of external constraints on policymaking, or citizens’ perceptions of such constraints, negatively impacts on citizens’ understandings of their domestic government’s responsiveness, providing indirect empirical support to Mair’s analysis as applied to citizens.

At the same time, however, these scholarships do not account for citizens’ understandings of political actors’ responsiveness and responsibility as such. Moreover, they depend on scholarly definitions of responsiveness and responsibility, assuming that citizens perceive them in the same manner. Arguably, this serves to conceal citizens’ unvarnished understandings. Yet, the few studies that investigate this more directly report results that support further research. First, perceived responsiveness of how well political actors meet citizens demands – not programmatic responsiveness, is consequential; especially in terms of citizens’ trust in political parties (Werner, 2016). Secondly, experimental research shows that voters’ relationships to parties are not based on promise-keeping (Werner, 2019). Thirdly, Linde and Peters (2020) suggest that responsiveness and responsibility do not need to be trade-offs, rather they can complement each other: by being responsive, governments can build a reservoir of goodwill, which can be used to implement responsible decisions (Linde & Peters, 2020). Thus, overlooking what citizens’ understandings of responsiveness and responsibility are on their own terms, how they might combine, and whether they result in a dilemma can result in spurious assumptions. This is why studying citizens’ understandings directly has increased pertinence.

### Our approach to citizens’ understandings

Following our abductive approach (see next section), our study begins from Mair’s inspired definition of political actors’ responsiveness and responsibility. Responsiveness relates to the representative role of national political actors and concerns their capacity and/or willingness to represent voters’ preferences (Lefkofridi & Nezi, 2020; Mair, 2013). In line with the delegate conception of representation (Pitkin, 1967), responsiveness is conceived as the direct link between voters’ policy preferences and political parties’ policymaking. Specifically, there are two main conceptualisations of responsiveness, both in line with the delegate conception, that differ according to the mechanism: responsiveness may be achieved through elections and promise-keeping, or thanks to a constant communication process, from voters to parties through public opinion (Werner, 2019). In the first case, the core objective of political actors is to fulfil their promises by implementing their pre-electoral programmes, consistent with the democratic style of ‘promissory representation’ (Mansbridge, 2003). In the second case, voters give constant input to their representatives and political actors respond to voters’ short-term demands as expressed through public opinion. In both cases, political actors listen to and then respond to citizens’ preferences (Mair, 2014). Responsibility pertains to the governing role of political actors and their compliance with accepted procedural norms and practices such as, in the European context, the constraints stemming from the EU (Mair, 2014, p. 588). Responsibility, thereby, involves an acceptance that, in certain circumstances, political actors’ hands will be tied.

From this perspective, we build on the qualitative turn in European studies, which provides a heuristic standpoint to analyse citizens’ discourses on the EU (Baglioni & Hurrelmann, 2016; Beaudonnet *et al.*, 2023; Belot, 2000; Díez Medrano, 2003; Duchesne *et al.*, 2013; Mercenier, 2019; White, 2011). We utilize elements of discursive institutionalism, which conceptualizes discourse as a transmission belt from elites to citizens (Schmidt, 2008), and citizens’ discourses which contain their perceptions, beliefs and understandings. From this, we conduct a constitutive analysis of citizens’ understandings of political actors’ responsibility and responsiveness in relation to the SEGEU with, ‘an examination of what th[ese] thing[s] (…) consist of and how [they] work’ (Cramer Walsh, 2012, p. 518; McCann, 1996). In this sense, we are not concerned with the frequency of specific understandings from subgroups of citizens across time, national and socio-economic divides, but whether citizens across relevant divides share understandings of political actors’ responsiveness and responsibility, what these understandings are, and how they are articulated as a common understanding.

From discursive institutionalism, we take the distinction between two types of ideas that discourses articulate: cognitive and normative (Schmidt, 2008). Discussions between citizens play an important cognitive role, helping them to make sense of political developments (Dimitrova & Kortenska, 2017). They provide the lens through which a given phenomenon is perceived, conceptualised and made sense of. We thus look at the cognitive frames citizens rely on to discuss political actors’ responsiveness and responsibility in the SEGEU. Generally, we define frames as socio-cultural resources citizens draw on to discuss and evaluate. A frame analysis is thus used to assess citizens’ understandings to the extent that ‘frames mediate the effect of micro and macro sociological factors on people’s attitudes towards European integration’ (Díez Medrano, 2003, p. 6). Discourses also embody normative elements. We look specifically at normative ideas in citizens’ understandings, and the extent to which they are debated across subgroups of citizens to constitute a norm. Following Thompson (1971), a norm emerges when a given understanding is not contentious and is held across relevant subgroups in political discussions (Vila-Henninger, 2020). Thus, investigating citizens’ discourses on the SEGEU offers insights into citizens’ understandings, both cognitively – the content of their understandings, as well as normatively – the shared principles that underlie their understandings.

## Data and methods of analysis: operationalising citizens’ understandings of political actors and the SEGEU

### Qualitative secondary analysis and comparative design

Secondary analysis, taken to mean ‘a research strategy which makes use of pre-existing […] research data for the purposes of investigating new questions or verifying previous studies’ (Heaton, 2004, p. 16) informs our approach. We perform a qualitative secondary analysis (Hughes & Tarrant, 2020) to enable us to study what, and how, research participants discuss specific political issues or phenomena.

Our secondary corpus includes three primary qualitative datasets of collective interviews generated in Belgium and France in 2005–2006, 2013–2014 and 2019 (see Table 1 and Appendix).^1^ Using 24 focus groups in Brussels, Oxford and Paris between October 2005 and June 2006, CITAE – Citizens Talking About Europe, generated data on citizens’ reactions to European integration (Duchesne *et al.*, 2013). Each group involved 4–7 participants selected to be socially close (working class, white collar, managers, and activists) but politically diverse. Here, we focus on the groups from France and Belgium comprised of 96 participants. In addition, we utilise Mercenier’s (2019) dataset focusing on young people’s perceptions of the EU and their relationships to politics. It includes 6 focus groups undertaken between November 2013 and May 2014, of 4–7 participants aged 16–26 and living in Brussels. The participants reflected the spatial segregation of Brussels, which was instrumental to recruiting interviewees with diverse socio-demographic features. The final dataset, RESTEP, was designed to study how citizens structure their discourses on Europe, and when and how European issues are politicised (Beaudonnet *et al.*, 2022). The research used 21 focus groups of different socio-economic groups in France, Belgium, Portugal, and Italy over four-months in 2019. Only those conducted in France and Belgium, comprising 14 focus groups and 69 participants, are used here.

Our main corpus is thus composed of three datasets that were generated at different points in time, in the same countries (Belgium and France), and thereby offering variation over time and policy contexts. They also display variation in support of the EU and EU institutions (Duchesne *et al.*, 2013). The extent to which domestic political elites accept and endorse the rules of the SEGEU also differ, with the French being particularly open to bypassing or contesting them (Hassenteufel & Palier, 2015). We focus on these two cases because we had access to focus groups conducted in more than one point in time. Each dataset offers socioeconomic and political variation as primary researchers selected participants based on both criteria. Moreover, the similarity of research questions – how citizens perceive and understand European integration – and methods in the original studies – cross-national qualitative research – supported a sufficient degree of cross-study comparability (Hughes *et al.*, 2023). While a great variety of arguments, justifications and explanations are present in these discussions on Europe, citizen discourses tend to converge on a coherent set of themes in which socio-economic-governance-related issues are well represented (Van Ingelgom, 2014). Apart from being the focus on this special issue, the SEGEU offers a relevant case to study how citizens’ understandings of responsiveness and responsibility could be analysed in their discourses. In this policy area, the constraints stemming from the EU have been salient over the last decade. The SEGEU is thus considered as an extreme case enabling the analysis of citizens’ understandings of political actors’ responsiveness and responsibility.

Because we are empirically interested in meaningful commonalities across time, place and socio-economic backgrounds, the remaining heterogeneity in primary research questions strengthens, rather than weakens, findings which emerge from otherwise independently generated and designed primary data sources. Importantly, in neither of the primary datasets were research participants prompted to discuss the SEGEU as such.

### Abductive analytical approach and operationalisation

We apply an abductive approach to qualitative analysis (Vila-Henninger *et al.*, 2022). Abduction was initially developed by Peirce (1934) to draw inferences that are oriented towards theory-building. It refers to the iterative process between theoretically surprising cases and tentative explanations (Tavory & Timmermans, 2014). As such, abduction is distinct both from deduction and induction but combines features of both. What sets abduction apart from a purely (ideal-typical) inductive form of inference is that the observed phenomenon does not contain an explanation in itself (induction), neither does it constitute a new case of an already known general rule (deduction) but is rather a combination of both. In this abductive perspective, our analysis is driven by the theorisation of citizens’ understandings of political actors’ responsibility and responsiveness in the context of the SEGEU. As a consequence, our study is not designed to offer a representative description of these understandings in different countries, time points and across socio-economic backgrounds, but rather to conceptualise citizens’ understandings that are anchored in both the existing literature and in our constitutive analysis.

In the first phase, we coded the primary datasets using a deductive team codebook (Vila-Henninger *et al.*, 2022). To operationalise research participants’ discussion of the SEGEU, we drew, first, from codes that cover policy domains as identified in the comparative agenda project. We included five policy codes – banking and finance, economy, employment, Euro, and social policies – that cover both the core of the SEGEU (e.g., ‘Euro’ or ‘economic’ policy) as well as its policy implications (e.g., ‘social policy’, ‘employment policy’). We adopted a broader understanding of the SEGEU than policy studies, because we analyse citizen discourses, not expert discussions, and in research settings where participants were not prompted (for further details on the coding of policy discussions see Dupuy *et al.*, 2022). Alongside the policy codes, we included a second code group that described the level of government where policies were discussed. Specifically, we included the code ‘Multilevel – EU’. We also relied on a code group based on Easton’s paradigmatic framework (1965) that depicts any actor, institution or action which connects citizens to their political system (for further details, see Vila-Henninger *et al.*, 2022). We included the ‘political actors’ and ‘regime institutions’ codes, indicating respectively when participants speak of any elected political actors invested with formal political power by their office or any governmental body or organisation.

In a second phase, we identified interview segments where political actors and institutions are mentioned in discourses on policies associated with the SEGEU. Code equation 1 retrieved quotations where any political actors were discussed in relation to policies included in the SEGEU. Code equation 2 retrieved quotations where any institutions were discussed as engaging in the SEGEU. Table 2 presents both code equations. This second step was instrumental in identifying relevant segments of discussions and performing data reduction (Table 2).

**Table 1.** Datasets included in our corpus.

Primary dataset	Primary data collection	Research topic	Type of interviews	Cross-national comparison*	Social composition	Number of groups/interviews and participants in our secondary dataset
CITAE (Duchesne *et al.*, 2013)	2006	Citizens’ reactions towards European integration	Focus groups	Belgium, France and the UK	Participants from varying socio-economic background	16 FG and 96 participants
Mercenier (2019)	2014	Citizens’ perceptions of the EU and their relationships to politics	Focus groups	Belgium	Young adults from different neighbourhoods with distinct socio-demographics	6 FG and 35 participants
RESTEP (Beaudonnet *et al.*, 2022)	2019	Citizens’ politicisation of EU issues	Focus groups	Belgium, France, Italy and Portugal	Participants from varying socio-economic background (high and low education levels) – including students	10 FG and 69 participants

**Table 2.** ATLAS.ti query tool abductive code equations.

Code Equation 1Political actors in EU socio-economic governance	Public Policy Code – Banking and Finance | Public Policy Code – Economy | Public Policy Code – Employment | Public Policy Code – Euro | Public Policy Code – Social policies AND Political actors code AND Multilevel code – EU level	36 quotes
Code Equation 2Institutions in EU socio-economic governance	Public Policy Code – Banking and Finance | Public Policy Code – Economy | Public Policy Code – Employment | Public Policy Code – Euro | Public Policy Code – Social policies AND Political actors code AND Multilevel code – EU level	96 quotes

In total, we retrieved 36 and 96 quotes from code equations 1 and 2 respectively. We then eliminated 29 redundant quotes that were coded with both ‘political actors’ and ‘regime institutions’. After a first round of analysis, we eliminated 32 false positives where typically, the ‘EU level’ code was present because of policy codes other than the ones of interest. In total, we analysed 71 quotes: 45 from the CITAE dataset, 14 from the Mercenier dataset and 12 from the RESTEP dataset.^2^ Quotes can range from a few sentences to a very lengthy discussion in a focus group – the shortest was 30 words (RESTEP dataset) and the longest amounted to 1751 words (CITAE dataset).^3^

In a third step, we coded for mentions of political actors’ and EU institutions’ responsiveness and responsibility. We began with Mair’s inspired-definition of responsiveness and responsibility as presented in section 2: responsiveness equates to political actors’ capacity, and/or willingness, to represent citizens’ preferences in EU socio-economic policymaking (Mair, 2013); whilst responsibility pertains to their compliance with institutional rules, especially those in the framework of socio-economic governance (Mair, 2014). We then inductively analysed the quotes to unveil possible alternative understandings of political actors’ responsiveness and responsibility in the context of the SEGEU.

## Empirical analysis

The following analysis illuminates participants’ discussions of political actors’ responsiveness and responsibility in order to study the cognitive frames they draw from. The analysis also shows how participants’ understandings were contested, so as to empirically capture their (un)shared components and assess the presence of related norms.

### Understandings of political actors’ responsiveness to citizens in the SEGEU

Across datasets, research participants frequently referred to the responsiveness of political actors or regime institutions when discussing the SEGEU (46 quotes). The principle according to which political actors should be responsive to ‘citizens’, ‘the people’ or ‘us’, was not questioned, rather it formed the baseline of discussions which queried its reality. Discussing whom political actors are responsive to in the real world, participants’ answers were straightforward: not ‘the citizens’. In a focus group with working-class participants in Brussels in 2005, political actors’ lack of responsiveness towards citizens was explicitly tied to absent political representation. While André explicitly acknowledges the principle of political representation, it is so far from his reality that he uses the conditional form ‘*if the elected representatives are our representatives …* ’.

Extract 1: Focus Group, Working Class, Brussels, 2005

André: I*f the elected representatives are our representatives^4^, there are some problems now in this society*. I propose to publicly fund elections because unfortunately, elected politicians are obliged to have a relationship with companies to finance … So, the ordinary people, the simple citizens don't have access to that. So: public funding. Secondly, the ideas of Switzerland that referendums take place more often because you see, for example in France, *all the politicians said ‘yes’ to the Constitution, ‘yes’ to Bolkestein and you see that there were 54 percent of French people who do not agree. So, there is a communication problem between elected politicians and citizens*. So, referendums or maybe another way to *have access to our politician because now they are not really our representatives unfortunately*.

André recalled that ‘all the politicians said, ‘*yes’ to the Constitution, ‘yes’ to Bolkestein*’. Even as citizens voted against the Constitution, the elites carried on with their plans, demonstrating a clear lack of responsiveness. This observation urged him to reflect upon the instruments necessary for politicians to listen to citizens and establish ‘*better communication*’. The problem of communication was also underlined by Jean-Michel, a participant in a sequential focus group organised with senior citizens in Grenoble in 2019. Discussing the ECB decisions to undertake quantitative easing, he explained that, *‘it's still a major event to have monetary policies like that, that's not up for debate. That is above all not put up for debate, it does not concern the people, it concerns the knowledgeable who know, who know moreover so well that things are getting better and better*’. Using irony, he denounced the absence of debate over the monetary policy at the EU level and how political elites exclude ‘*the people*’ from the discussion. He considers that the disconnection between the ‘*knowledgeable who know*’ and the people results in detrimental decisions.

Political actors are clearly constructed as a differentiated category from ‘*the ordinary people, the simple citizens*’ as André puts it (extract 1); this construction of two distinct groups grounds the discussions of political actors’ responsiveness. This is illustrated in extract 2, by participants in a focus group for young people living in Jette, a Brussels neighbourhood in the middle band of the socioeconomic spectrum. In their discussion of the European Coal and Steel Community and who pushed for it, they straightforwardly place politicians and ‘the people’ in opposition.

Extract 2: Jette (Brussels), 2014

Gabriel: In the end, whose decision was it?

Moderator: What decision?

Gabriel: The European Union. *Was it the people?*

Yusef: No, it was after the war, I think.

Lucie: It was just the first six countries, wasn't it?

Gabriel: *Was it the people or anyone else’s?*

Lucy: *The leaders of those six countries then?*

Catherine: *Originally, I think it was an alliance between them to avoid trouble, they said: well, that's it.*

Gabriel: Between whom?

Catherine: The leaders of the countries.

Nour: *But it's a good question that you raise in the sense that we are Europeans, we are in the European Union, but we don't even know who took these decisions. Is it the people? Is it, I don't know, the leaders?* It's still enigmatic.

Lucie: *But at the beginning it was purely economic because it was the coal and steel community. It was first for the money and the transfer of hardware. But afterwards, about the European Union,* I don't know.

Commenting on who decided to create the EU, Nour is puzzled by her observation that while she and the other participants are Europeans, they do not know who took the decision. She wonders if it was ‘the people’ or political leaders, with both groups understood as mutually exclusive. Catherine suggests that the leaders of the founding countries made the decision ‘*between them*’, in exclusion of other considerations and actors. In this discussion, participants felt estranged from the decision to create the EU. Citizens’ inputs, and the possibility of political actors’ responsiveness to citizens are quickly and un-contentiously dismissed. Other discussions converge on the understanding that elites made the decision and imposed their ‘*will*’ on citizens. For instance, a focus group with working class participants in Paris in 2005 wondered who decided to create the Euro. They too oppose politicians to ‘*the populations*’. As Gerald says, *‘they wanted it*’; Zahoua concurs by adding that politicians forced their decisions on citizens – ‘*they imposed it*’.

In discussions of political actors’ responsiveness, the opposing categories of political elite and citizens, share a common feature, however: both are defined as large and indefinite groups. Specifically, political actors are discussed without any mention of their ideological leanings or party-political affiliations with a few exceptions only. Similarly, citizens are conceived of as one group, with virtually no references to different interests, ideological preferences or socio-demographic characteristics. Thus, citizens are neither conceived of as voters, nor in relation to their choice of party. This finding challenges the definition of responsiveness as ‘promise keeping’, as argued by the mandate theory of democracy. Rather, it aligns with recent research asserting that citizens do not share the normative assumption underpinning political representation that political actors should keep their electoral promises (Werner, 2019). Instead, political actors’ responsiveness is understood as something broader. They are expected to respond to what the citizenry wants or needs, rather than their particular voters. This understanding is close to the normative demand for representatives to consider the needs of all citizens (Werner, 2019). In this sense, political actors are perceived as trustees, entrusted with decision-making capabilities through elections and are then tasked to decide on policy solutions that work best for citizens in general (Pitkin, 1967).

### Understandings of political actors’ responsiveness to market forces in the SEGEU

Across the dataset, alongside political actors’ lack of responsiveness to citizens’ preferences in the context of the SEGEU, participants also shared the understanding that political actors are responsive to another group: market actors and *‘the economy’*. Extract 3, from a focus group with working class participants in Brussels in 2005, illustrates the perception of political actors’ unequivocal responsiveness to the market and the economy in the context of the SEGEU. Farouk presents the Bolkenstein directive in a stylised manner, drawing from a neoclassical understanding of economics whereby market forces prevail over national economic interests since, ‘*the worker from another country comes to work in Belgium and pays his taxes in his own country*’. Farouk observed that ‘*the Bolkenstein law*’ was opposed by the French precisely because it was tilted against the common interest of a country’s citizens, as foreign workers ‘*pay the minimum of taxes here, they pay their taxes in their own country*’.

Extract 3: Working Class, Brussels, 2005

Farouk: And now the market is investing the minimum to earn the maximum. That's the point of view of the current market (…) To use the minimum amount of labour to get more money (…). It's like the Bolkenstein law. The worker from another country comes to work in Belgium and pays his taxes in his own country. I'll say that it's the opening up of the market, it's the market. That's what they want to do with the opening of borders, that a worker comes, a company comes, they open their company. Here already, a German truck company, they come to work in Belgium with their own cash-in-transit vehicles and everything and they pay the minimum of taxes here, they pay their taxes in their own country. *That's why the French voted no. When we asked them about Europe and everything here we weren't given a choice, well, I'm going to say that I have a bit of a trade unionist attitude, I'm a trade union delegate.*

In a focus group organised with managers in Paris in 2005, Gabriel also highlighted that political actors are responsive to ‘*the market*’ as ‘*elected politicians are obliged to take the market into account when they deal with a situation.*’

Importantly, the participants concurred that the responsiveness to market actors is the result of political actors’ own-decision making. Political actors thus enjoy agency. Discussing a proposal to lower the VAT on restaurant bills, Gabriel, stressed that ‘*The power in this case is all the finance ministers and so on, etc., it's really people who have been elected and who have the power to do or not to do something about this specific problem*’. Similarly, Farouk (extract 3), also underlines that the Bolkestein directive represents ‘*what they [the elite] want to do with the opening of borders*’. For him, the decision to favour ‘the market’ over citizens’ preferences was not only the case of the Bolkenstein directive and the French, but European integration more generally, where European citizens ‘*weren't given a choice*’. There were many other references to political actors’ deliberate decision-making, such as Catherine, who in the discussion on the Coal and Steel community in extract 2, suggested, ‘*Originally, I think it was an alliance between them to avoid trouble, they said: well, that's it*’.

While participants recognised that political actors made the consequential decision to be responsive to market actors in the context of the SEGEU, they also importantly observed that political actors were left with little choice in determining the course of action. The lack of alternative is explained by the dominance of market actors. As Gabriel put it, ‘elected politicians are obliged to take the market into account when they deal with a situation’; just like André who observed that political actors ‘*are obliged to have a relationship with companies, finance*’. This understanding of responsiveness is related to participants’ sense-making of these actors’ responsibility, with the former acknowledging that the latter has to take into account the claims of audiences other than the national electorate, including international markets (Bardi *et al.*, 2014, p. 237). In seeking to act responsibly, political actors are constrained by actors other than their electorate (Mair, 2014).

Overall, participants from different countries, various social backgrounds and at distinct points in time, unambiguously support the principle that political actors should be responsive to ‘the citizens’ in the context of the SEGEU. At the same time, they agree that they are not responsive to them, but rather to market actors and ‘the economy’. These observations reflect a clear-cut and outspoken distinction between ‘us’, the citizens – a large and indefinite category that is distinct from voters – and ‘them’, the political elite. Importantly, participants claim that political actors made the decision to be responsive to market actors and thus possess some agency but, by seeking to act responsibly, this leaves them with no other choice than to comply with market actors’ preferences. The cognitive frame they rely on to understand political actors’ responsiveness in the SEGEU is that of political actors’ agency and choice.

### Understandings of political actors’ responsibility in the SEGEU

Research participants did discuss the responsibility of political actors and institutions, albeit less frequently than (non)responsiveness (18 quotes). Across the data, there were some disagreements about what political actors’ responsibility was in the context of the SEGEU. Many participants understood responsibility as the compliance to agreed institutional rules stemming from European integration. Extract 4, from a focus group with managers in Brussels in 2005, neatly illustrates this.

Extract 4: Managers, Brussels, 2005

Fabio: *So, well, now is cooperation a real feeling that is felt by the population or is it a de facto cooperation that has been imposed by force of circumstance?*

Judith: Well, yeah, that's it.

Bruno: A little bit by force of circumstance but I think it's easy for nations to give up part of their power for something superior when they weren't necessarily obliged to.

Fabio: No, it's not easy, and it's the proof that the European integration is not progressing as we had hoped.

Bruno: It's obvious.

Fabio: *But this delegation of sovereignty was made again because we didn't have much choice and the states were dragged into a spiral that was beyond them.*

Bruno: Of course

Judith: What is it then that is beyond us?

Fabio: *Well, it's simply by pooling certain sectors, first the economic sectors, little by little: we were forced to go beyond that because imbalances were appearing, at the same time we had to pool policies; then, well, the economy, as it affects all sectors.*

Here, the participants explicitly discussed the ‘*cooperation*’ between EU member-states as constituting a ‘*delegation of sovereignty*’ that has gradually been taken out of their hands. They both highlight the initial political decision to create the common market as ‘*it’s easy for nations to give up part of their power for something superior when they weren't necessarily obliged to*’; but they also insist on political actors’ current lack of agency. They understand it as the outcome of external pressures associated with the process of integration, since by ‘*pooling certain sectors, first of all the economic sectors, little by little: we were forced to go beyond that because imbalances were appearing*’. While political actors are discussed in vague terms, they are referred to as ‘Belgium’ or ‘Germany’ or ‘France’, rather than ‘the elected politicians’ or ‘the politicians’, indicating that participants specifically discuss national political actors and suggest that the pressures come from the supranational level in line with Mair’s argument.

However, a few participants disputed this understanding. They contested the principle of responsibility as compliance to European rules by highlighting the detrimental outcomes it brings to European citizens. Acting responsibly in that sense prevents political actors from achieving the common good. In Extract 5, from a focus group conducted with white collar workers in Grenoble in 2019, the discussion between Delta and Golf about the principle of responsibility defined as compliance to European rules is heated. While they agree that national governments face institutional constraints stemming from the EU, they disagree on whether there are good reasons to accept them and act ‘responsibly’.

Extract 5: White Collars, Grenoble, 2019

Delta: I have more of a problem with Europe. In particular, the treaties, for example *t*he* fact that we can't go into debt beyond 3% of GDP, the fact that we can't modify these treaties, that the European Central Bank is independent, a sort of chicken without a political head that dictates all the monetary policies of each country, in fact. And it turns out that these are the levers that are vital for national public policies which can influence unemployment, all the economic activity of a country, in fact, yes*. With the government, well with the governance of Europe mainly, yes.

Moderator: Does everyone agree with that?

Golf: *No, because if they do that, it's because there are good reasons. Like the 3% is to prevent countries from getting into too much debt and to avoid that it results in problems for the 27, and that it destabilises the EU.*

Delta: *Well, that's one way of looking at it, but in fact it's mainly to increase competition and, finally, to participate in the dismantling of public services because, basically, if a state can't exceed that amount, it can't invest in public spending*.

Delta makes the argument that the rules of the Growth and Stability Pact (GSP) result in national governments’ decision-making capacity being hollowed out, turning them into agency-less actors who cannot make appropriate choices to fight unemployment or foster the country’s ‘*economic activity*’. Golf’s defence of these rules is grounded in the understanding that complying with them is the right thing to do to avoid ‘*problems for the 27*’. Delta stresses Golf’s common-sensical argument – ‘*it’s one way to look at it*’, and further elaborates on the negative consequences of the GSP rules: they hinder national governments ability to ‘*invest in the public services*’. He therefore makes clear how the compliance to the institutional rules of the GSP is a political choice made by national governments that yields negative consequences. Concomitantly, he elaborates on an alternative understanding of political actors’ responsibility whereby being responsible means acting towards achieving the common good. He associates it with ‘*public spending*’ and ‘*public services*’. Importantly, Delta’s understanding of responsibility restores not only political actors’ agency, but also their choice-making as they can choose to counteract ‘*the dismantling of public services*’ and to ‘*invest in public spending*’.

In a focus group with activists in Brussels in 2005, Charles-Henry and Vinciane also engaged with the principle of responsibility as compliance to EU rules. Vinciane challenged this principle based on its outcomes. ‘*Without a strong taxatio*n’, Vinciane argues, policies cannot achieve ‘*solidarity*’. Charles-Henry responds to this argument by stating that ‘*we can support solidarity in a responsible way*’. In this sense, achieving the common good, by associating either with public services or the principle of solidarity, is defended as an alternative definition of political actors’ responsibility in the context of the SEGEU. It is grounded in an understanding of responsibility where political actors have agency and can make choices.

Overall, many participants understood political actors’ responsibility in the context of the SEGEU in terms that are close to Mair’s definition: they point to the prevalence of institutional rules stemming from European integration, as well as the interdependence of national governments. However, this does not go unchallenged and an alternative understanding is elaborated on – even if rarely, that defines responsible actors as striving to achieve the common good. However, in our data, the principle of responsibility is not taken for granted and there is no agreed norm for how participants understand political actors’ responsibility in relation to the SEGEU. Nonetheless, whilst they disagreed, participants relied on a shared cognitive frame to discuss responsibility, which is that of political actors’ capacity for agency and choice.

### Being responsive and responsible: a dilemma?

The question that remains is how far did participants perceive the responsiveness and responsibility of political actors in the SEGEU to be dilemmatic, and if so, in what terms? In our data, there are only a few discussions where political actors’ responsiveness and their responsibility are discussed contiguously. This strongly chimes with two of our empirical results: first, participants do not consider that political actors are responsive to them, so it makes sense that the dilemma is not prevalent; second, they question the principle of responsibility, which logically constrained their discussions of the dilemma. However, there are still instances (10 quotes) where participants discuss political actors’ responsibility and responsiveness side by side. The dilemma is structured along the line of political actors’ agency and choice, thereby following the cognitive frame participants rely on to discuss both responsiveness and responsibility.

In a focus group organised with pensioners in 2019 in Grenoble, participants discuss the case of Tsipras, a rare instance where a politician is singled out by name. Jean-Michel and Sophie are outraged by his ‘submission’ to European rules and his breaking of electoral promises, denouncing his lack of agency and constrained choices; while Nadine accepts that he could not do otherwise and legitimises his decisions.

Extract 6: Senior, Grenoble, 2019

Jean-Michel: I mean, the Euro killed their tourism and killed their agriculture.

Sophie: They sold to the governments … 

Jean-Michel: *Tsípras got elected saying he was going to solve the problem, and finally he was subjected.*

Nadine: *He couldn't do otherwise*.

Sophie: Well yes.

Roger: Well, that's obvious.

Jean-Michel: *Well, in any case, he made a lot of noise with his mouth and not much with his hands.*

Roger: Well it's money, eh. Not the currency!

Sophie: Sure, it's spectacular.

Nadine: *Well he didn't have the …* 

Jean-Michel: *It's bordering on election fraud, what he did.*

Sophie: Yes.

Nadine: *Yes, but when Europe puts a knife to your throat, what do you do?*

Jean-Michel: You're right, you're right.

Sophie: No, but we were talking about incarnation earlier and it's true that *T*s*ípras is special because he really sold something that he didn't keep. But despite everything, he continued on his way, he wasn't debunked*.

Even if Jean-Michel describes what Tsipras did as ‘*bordering on electoral fraud*’, he still agrees with Nadine when she asks ‘*Yes, but when Europe puts a knife to your throat, what do you do?*’. They both recognise that Tsipras has agency, albeit bound by EU rules. In times like the economic crisis in Greece, the increasing pressure for responsibility, understood as compliance with EU rules, goes hand in hand with the perceived inability of political actors to listen and be responsive to voters: their responsible but unresponsive policies are thereby deemed justified. This exemplifies how participants perceive that in specific contexts, responsibility can take precedence over responsiveness (Laffan, 2014; Lefkofridi & Nezi, 2020).

In other discussions however, political actors’ responsiveness and responsibility in the SEGEU are discussed concurrently, but without the construction of a dilemma or a tension between the two. This is the case in a focus group organised with young people in 2014 in Ixelles, a comparatively rich neighbourhood in Brussels.

Extract 7: Ixelles, 2014

Louis: There will be no change in the institutions, the organs of power that are the institutions. *You take the IMF or the European Commissariats, they are outside of any democratic direction. You are asked to vote for the European Parliament. The European Parliament can give guidelines. You can imagine how much that matters when you are a European Commissioner, a guideline from the European Parliament. So, all these organs of power are going to say to Belgium ‘you are saving ten billion today to service your debt’. They have no intention of changing this. You can't ask institutions to change. They are not going to change by themselves. It's me who doesn't understand. It's the institutions, yes, in Europe,* *but do you think the IMF will propose to dissolve itself? To leave more room for Belgium?*

Isabella: *No, I think that I was just talking about the Belgian state which faces constraints, could not do what it wanted. So, it was in a union yes. It's simple. For example, you are part of a group, you can't do what you want to do. You have to ask others.* You're on a board of directors, you have to ask the others, you can't lead on your own. So that's what I mean.

When Louis discusses the hurdles to institutional change, he specifically refers to situations where governing political actors must act within accepted rules of monetary and budgetary policy. He cites the European Commission and the IMF as institutions that constraint ‘Belgium’ from autonomous decision-making. Louis, just like Jean-Michel and Nadine in the previous extract, considers that responsibility prevails in political actors’ decision-making while responsiveness is side-lined. Nevertheless, there is no tension or dilemma, here. While Isabelle agrees that Belgium is constrained by supranational institutions, she nuances the negativity of Louis’ assessment. She normalises the situation that Louis presented in a vivid, negative tone, by suggesting that because Belgium is part of ‘*a group*’, it is only fair that other members of the group have their say. She therefore accepts, as Mair stated, that ‘in certain areas and in certain procedures, the leaders’ hands will be tied’ (2013, p. 158).

Overall, participants recognise that there is a growing gap between responsiveness and responsibility – or between what citizens might like political actors to do and what they are obliged to do (Mair, 2014), but the dilemma is less obvious to them. Only a few participants recognise a dilemmatic relationship between political actors’ responsiveness and responsibility, and these discussions close the dilemma with the observation that compliance to EU rules and constraints from intergovernmental cooperation undeniably prevails over political actors’ responsiveness to citizens.

## Discussion and conclusion

This article has investigated citizens’ understandings of political actors’ responsibility and responsiveness in the context of the SEGEU. It is based on a qualitative secondary analysis of focus groups, with participants from various socio-economic backgrounds in Belgium and France, and undertaken in the mid-2000s, the mid-2010s and 2019. Although complex, participants were not prompted, and discussed multiple issues from various angles. Their discussions were not limited to the benefits or costs they themselves, or their country, may derive from their membership to the EU, nor to an assessment of policy performance.

First, while we do not claim to make a causal argument on the changes around Next Generation EU, we report that prior to its adoption, participants from all groups concurred that political actors were not responsive to citizens’ needs; and deemed this highly problematic. They understand politicians’ responsiveness as addressing the needs of citizens in general, and their discussions on responsiveness very rarely mention electoral promises and political parties. This understanding is endorsed across the data, which supports our analysis that there is a norm relative to the lack of political actors’ responsiveness to citizens.

Secondly, participants did not all agree that responsibility as a principle, which most understood as compliance with external actors’ requirements, should guide political actors’ policymaking in the context of the SEGEU. A few even elaborated a distinct understanding of political actors’ responsibility as seeking the common good. This alternative understanding articulates notions of good government as attending to citizens’ basic and shared needs. However, this is distinct from ‘responsive responsibility’ (Crespy *et al.*, 2024) as participants’ discourses, in general, with a few exceptions, reflect no closing of the dilemma between responsiveness and responsibility, something which is crucial in ‘responsive responsibility’. On the contrary, the alternative understanding of responsibility some participants discussed, emerged from the contested nature of the norm of responsibility and the conflictual definitions participants relied on; one that is developed alongside the understanding that political actors are not catering to citizens’ needs and preferences.

Thirdly, while in all three primary datasets, most participants framed responsibility and responsiveness in terms that are close to Mair’s definitions, their understandings did not result in the elaboration of the dilemma consistent with them. The reason is that politicians’ responsiveness is so clearly missing. Consequently, participants suggest that there is a growing gap between political actors’ responsiveness and responsibility (Mair, 2014, p. 593).

Fourthly, our analysis has highlighted how participants drew on a shared cognitive frame to discuss political actors’ responsiveness and responsibility in the context of the SEGEU – regardless of country, time, and socio-demographics. This cognitive frame centres on political actors’ agency and choice. Most participants concurred in their observation that political actors made the *deliberate decision* to be responsive to ‘the market’ and ‘the economy’, and consequently had little choice left when selecting courses of action as political actors are understood either as transmission belts of market actors’ preferences or as fully dominated by multinationals and economic elites. In contrast, in the discussions about their responsibility, participants queried whether political actors *could* make the decision to follow or disregard the institutional rules stemming from the EU, and if they *could* make alternative choices in the context of the SEGEU. Understandings of both political actors’ responsiveness and responsibility are thus tied to actors (lack) of agency and choice. These results are strengthened by our case selection, as Belgium and France exhibit variation on citizens’ support to the EU and elites’ compliance with SEGEU commitments. In that respect, our analysis offers empirical support for Hay’s four-pillared conception of the political as entailing ‘ … *choice, the capacity for agency, (public) deliberation, and a social context*’ (2007, p. 65 – italics in original). What makes Hay’s conceptualisation attuned to account for citizens’ understandings is the reasoning that politics occurs only in situations of *choice* (Hay, 2007, p. 65; see also Wood & Flinders, 2014) and where ‘actors possess and display the *capacity for agency*’ (2007, p. 67, our emphasis). As the possibility of political choice is crucial to understand citizens’ connections to their national political system (for an analysis at the national level, see Hobolt & Hoerner, 2020), political actors’ partial lack of agency and choice in the context of the SEGEU, yields consequences in terms of how European citizens understand their relations to the political system (Dupuy & Van Ingelgom, 2019).

Following our abductive approach, and its theory-building orientation, we contend that these empirical results have significant implications for our conceptualisation of responsiveness and representation from a citizen perspective. Our findings indicate that participants’ understanding of political actors’ responsiveness is at best only loosely related to political parties, elections, or direct communication between parties and voters. Across datasets, participants did rarely discuss electoral promises, or whether or not politicians kept them, as a means to ground their understanding of responsiveness. Neither did they mention the necessity to have, strengthen or alter in any way, a direct communication between parties and voters. Importantly, participants did not think of themselves, or other groups of people, as voters, or related to a preferred party or party choices. Rather, they used the general category of ‘citizens’ that they placed in opposition to the equally large and indistinct category of ‘politicians’. As anticipated by Mair, parties appear to have failed to persuade voters to accept them as crucial features of political life (Mair, 2014, p. 581). Thus, if we are to theorise responsiveness from the perspective of citizens, the delegate conception of representation (Pitkin, 1967) provides little guidance as it does not reflect how citizens understand it. In line with emerging research (Werner, 2019), we suggest that a trustee type of relationship, whereby citizens entrust political actors with solving problems and attending to their needs, resonates more closely with citizens’ perspective. In this respect, the key mechanism of responsiveness is political actors’ dealing with issues and solving problems that citizens face (Amara-Hammou, 2023). When the perspective of citizens is adopted, circling back to the article’s introduction, our results suggest that the dilemma between political actors’ responsibility and responsiveness is not likely to be historically contingent. In Mair’s own words, ‘we have a situation in which the malaise is pathological rather than conditional’ (Mair, 2014, p. 593).

## Supplementary Material

Supplemental Material

## Notes

1 The distinction between primary and secondary data is based on the relationships between the researcher who generated a data set and the researcher who is analysing it: ‘If the dataset in question was collected by the researcher (or a team of which the researcher is a part) for the specific purpose or analysis under consideration, it is primary data. If it was collected by someone else for some other purpose, it is secondary data’ (Boslaugh, 2007, p. 1).

2 Note that the CITAE dataset includes 16 focus groups, while the Mercenier dataset is composed of 6 focus groups and the RESTEP project includes 10 focus groups. The higher number of quotes from CITAE could thus be partly explained by the larger amount of data it features. In addition, in the CITAE dataset, the scenario of each focus group included a question on how the power should be distributed in Europe, which directed the discussion towards political actors and institutions.

3 The total length of the 71 quotes that were analysed is of 27917 words and thus the average length of a quote is of 393 words (428 for CITAE, 410 for Mercenier and 240 for RESTEP).

4 Underlined text segments identify political actors, regime institutions and SEGEU-related policies that are discussed by participants. Segments in italics identifies where responsiveness and/or responsibility are discussed.
