# Supplementary material for: Neither responsive, nor responsible? Citizens’ understandings of political actors’ responsiveness and responsibility in the socio-economic governance of the EU
Source: J Eur Public Policy. 2023 Dec 23;31(4):1126–52. doi: 10.1080/13501763.2023.2288236 (PMC11523917; doi:10.1080/13501763.2023.2288236)
Supplement: Supplemental Material [file RJPP_A_2288236_SM2657.docx]

**APPENDIX 1**

**PRESENTATION OF DATASETS**

**The Citizens Talking About Europe Dataset**

The CITAE data set is based on data gathered from focus groups, conducted in Oxford, Paris, and Brussels between January and June 2006 (Duchesne and al., 2013). It is based on eight collective discussions (focus groups) involving four to six participants selected as socially close but politically diverse. In sum, these focus groups were conducted with British citizens from different socio-economic contexts (working class, white collars, managers and activists). Each focus group discussion lasted about three hours, and was organized around five questions only – in order to leave enough room for participants to lead the discussion in the directions that would interest them most. The five questions tried to cover different aspects of European integration: identity, institutions, benefit and membership, as well as political sophistication. The questions posed were as follows: 1. What does it mean to be European? 2. How should we distribute the power in Europe? With suggestions, in order to structure the discussion, and requests to discuss what would be desirable or undesirable about power resting with the Nations, with Experts, with MPs or with the Market (i.e. left to market forces). Then there was a PAUSE for refreshments. 3. Who profits from Europe? This question was posed to sub-groups, and their written responses were then discussed by the whole group; 4. For or against Turkey’s entry into the European Union? This discussion was preceded by a yes or no vote by each participant individually. 5. For or against Turkey’s entry into the European Union? This time participants were asked to answer this question from the point of view of political parties from the country in question – list of parties was suggested by the moderator. This question both cooled any conflict as answering it was a cooperative enterprise; and also serves as a kind of test of political knowledge.

Main reference: Duchesne, S., Frazer, E., Haegel, F. & Van Ingelgom, V. (2013). *Citizens’
Reactions to European Integration Compared. Overlooking Europe.* Palgrave McMillan.

**Heidi Mercenier’s dataset**

Heidi Mercenier’s dataset consists of 6 focus groups with young people (16-26 years) living in different neighborhoods belonging to the Brussels Capital Region and also reflecting variation in economic affluence. The focus groups have been organized in 2014 with a total of 35 young citizens. The original study had been designed to better understand the perception of the EU by young citizens through the analysis of the citizens’ discourses underlying the legitimacy and political credibility of the EU. Each focus group featured six questions. Each session started with the question on the most important problem (question 1) and a discussion on who was supposed to resolve these problems (question 2). In a second block, participants were asked about their perceived distance to different levels of power represented by a series of photographs (question 3) and what EU meant to them (question 4). In a third and last block on identity and belonging, questions were about identifying “groups of persons” to which participants felt belonging (question 5) as well as indicating different entities on different levels of power they felt attached and explaining the reasons thereof (question 6). Each focus group took about three hours with a 10 minutes break after question 1 and 2, i.e. after around 1 hour.

Main reference: Mercenier, H. (2019). *« C’est compliqué ! » : L’Union européenne vue par des jeunes Bruxellois Contribution à l’étude des rapports des citoyens à la politique.* Doctoral thesis, Université Saint-Louis – Bruxelles.

**RESTEP dataset**

These data were collected by RESTEP (RÉSeau Transatlantique sur l’Europe Politique), an international research network that involves researchers from 10 European and Canadian universities. Similarly to the other primary datasets, this data were designed to advance the understanding of citizens’ relations to European integration. Altogether, 21 focus groups were organized in 4 EU members states (Belgium, France, Italy and Portugal) during the EP election year 2019. Focus groups were designed so as to cover 5 different socioeconomic backgrounds (Seniors; Student; Young unemployed; Young without diploma; White-collar workers). Each focus group featured 5 to 9 participants and some of them (Seniors) gathered 3 times over a 4-month-period from March to June 2019. Out of the 21 Focus Groups, we used those 14 organized in France (Grenoble) and Belgium (Louvain-la-Neuve).

Main reference: Beaudonnet, L., Belot, C., Caune, H., Dupuy, C., Houde, A., Le Corre Juratic, M .. & Van Ingelgom, V. (2022). Studying (De-)Politicization of the EU from a Citizens Point of View: A New Comparative Focus Group Study. *Politique européenne*, 75, 100-122.

**APPENDIX 2**

**PRESENTATION OF PARTICIPANTS FOR EACH DATASET**

**1. CITAE Focus Group participants**

Source: Duchesne, S., Frazer, E., Haegel, F. & Van Ingelgom, V. (2013). Citizens’ Reactions to European Integration Compared. Overlooking Europe. Palgrave McMillan.

| **(Nick)**  **name** | **Sex** | | **Age** | | **Education** | | **Profession** | | **Left right** | | **Vote** | | **Referendum** | | **EU belonging** | | **Identity** | **Origin** |
| --- | --- | --- | --- | --- | --- | --- | --- | --- | --- | --- | --- | --- | --- | --- | --- | --- | --- | --- |
| **PAR Working Class 1** | | | | | | | | | | | | | | | | | | |
| Albert | M | 42 | | Brevet/BEPC | | Naturopath (unemployed) | | 5 | | NV | | NV | | G | | World | | White |
| Ghislaine | F | 26 | | Brevet/BEPC | | Care assistant | | 4 | | L. Jospin | | NV | | G | | NE | | Afro- Caribbean |
| Geoffrey | M | 33 | | CAP ou BEP | | Print worker | | 5 | | NV | | N | | NGNB | | NE | | White |
| Lionel | M | 42 | | Brevet/BEPC | | Security officer | | DK | | O. Besancenot | | N | | G | | EN | | White |
| Yasmina | F | 35 | | Brevet/BEPC | | Home-maker | | DK | | NV | | NV | | B | | NE | | Maghreb |
| Habiba | F | 41 | | Bac general | | Home-maker (and secretarial work for family business) | | 4 | | L. Jospin | | No | | NGNB | | Other | | Maghreb |
| **PAR Working Class 2** | | | | | | | | | | | | | | | | | | |
| Jean-Marie | M | 53 | | Brevet/BEPC | | Auto mechanic (unemployed) | | DK | | C. Lepage | | No | | NGNB | | NE | | White |
| Cédric | M | 38 | | Bac général | | Charge nurse | | DK | | L. Jospin | | Nul | | NGNB | | NE | | White |
| Jeannette | F | 25 | | Brevet/BEPC | | Care worker in training (numerous jobs) | | 6 | | NV | | NV | | G | | Other | | Africa |
| Zahoua | F | 45 | | Brevet/BEPC | | Medical secretary (unemployed) | | 1 | | NV | | NV | | NGNB | | N | | Maghreb |
| Margot | F | 40 | | CAP ou BEP | | Lorry driver | | DK | | C. Lepage | | NA | | B | | N | | White |
| Gérald | M | 37 | | CAP ou BEP | | Heating engineer | | DN | | NV | | NV | | G | | N | | Other |
| **PAR White Collars 1** | | | | | | | | | | | | | | | | | | |
| Laetitia | F | 23 | | Bac +2 | | Sales engineer | | 6 | | J. Chirac | | NV | | B | | N | | White |
| Magali | F | 28 | | Bac +2 | | Receptionist/telemarketing | | DK | | J. Chirac | | NA | | NGNB | | NE | | White |
| Victor | M | 30 | | Bac +2 | | Higher technician, logistics | | 2 | | N. Mamère | | N | | G | | E | | White |
| Patrice | M | 33 | | Bac tech/pro. | | Butler | | DK | | NV | | NV | | NGNB | | NE | | White |
| Hadia | F | 36 | | Bac +3 à +5 | | Project leader, advertisement (unemployed) | | 3 | | NV | | NV | | G | | NE | | Maghreb |
| Clélia | F | 24 | | Bac+2 | | Receptionist/illustrator | | 5,5 | | NA | | NV | | ? | | ? | | White |
| **PAR White Collars 2** | | | | | | | | | | | | | | | | | | |
| Pablo | M | 43 | | Bac tech/pro. | | Secretarial work (unemployed) | | 7 | | J. Chirac | | No | | G | | N | | Other European |
| Samira | F | 26 | | Bac +2 | | Restaurant manager | | 5 | | L. Jospin | | No | | B | | N | | Maghreb |
| Paul | M | 38 | | Bac, formation medical sales representative | | Medical sales representative | | 5-6 | | NA | | No | | NA | | NA | | European |
| Aline | F | 41 | | Bac +2 | | Sales engineer (unemployed) | | 6 | | J. Chirac | | NV | | B | | E | | White |
| Martin | M | 46 | | Bac+2 | | Graphic designer (unemployed) | | 3 | | L. Jospin | | Y | | G | | NE | | White |
| **PAR Managers 1** | | | | | | | | | | | | | | | | | | |
| Franck | M | 35 | | Bac +3 à +5 | | IT professional | | 7 | | J. Chirac | | Y | | G | | EN | | White |
| Inès | F | 39 | | Bac +2 | | Fashion designer | | 7 | | J. Chirac | | N | | G | | NE | | White |
| Fabienne | F | 26 | | Doctorat | | Doctoral student | | 3 | | M.-G. Buffet | | N | | B | | World | | White |
| Gabriel | M | 59 | | Bac +3 à +5 | | Printing advisor | | 3 | | L. Jospin | | Y | | G | | NE | | White |
| Toufik | M | 24 | | Bac +3 à +5 | | Engineer | | 4 | | NV | | NV | | G | | NE | | Maghreb |
| Serge | M | 42 | | Bac +3 à +5 | | Chartered accountant | | 5 | | L. Jospin | | N | | B | | EN | | White |
| Céline | F | 31 | | Bac +3 à +5 | | Translator | | 4 | | NV | | N | | G | | NE | | White |
| **PAR Managers 2** | | | | | | | | | | | | | | | | | | |
| François | M | 42 | | Bac +3 à +5 | | Computer/logistics manager | | 9 | | J. Chirac | | Y | | G | | NE | | White |
| Michel | M | 46 | | Bac +3 à +5 | | Management controller | | 6 | | J. Chirac | | N | | NGNB | | EN | | White |
| Patrick | M | 38 | | Bac +3 à +5 | | Tax law specialist, civil servant | | DK | | J. Chirac | | Y | | G | | EN | | White |
| Jean-Paul | M | 60 | | Doctorat | | Math professor, university | | 6 | | NV | | Y | | NGNB | | NE | | White |
| Louis | M | 49 | | Bac +3 à +5 | | Teacher/photographer (ex-marine officer) | | 3 | | L. Jospin | | N | | NGNB | | E | | White |
| Stanislas | M | 50 | | Bac +3 à +5 | | Information officer (medical) | | 5 | | J. Chirac | | Nul | | G | | NE | | White |
| **PAR Activists 1** | | | | | | | | | | | | | | | | | | |
| César | M | 35 | | Bac +3 à +5 | | Lawyer (unemployed) | | 6 | | J. Chirac | | Y | | NGNB | | Other | | Afro-Caribbean |
| Charles | M | 21 | | Bac +3 à +5 | | Student (engineer) | | 8 | | NV | | N | | G | | NE | | White |
| Cheik | M | 40 | | Bac tech/pro | | Municipal agent | | 6 | | J. Chirac | | NV | | G | | NE | | Maghreb |
| Pierre-Antoine | M | 23 | | Bac +3 à +5 | | Communications manager (party) | | 4 | | F. Bayrou | | Y | | G | | NE | | White |
| Déborah | F | 30 | | Doctorat | | Doctoral student | | 5 | | L. Jospin | | Y | | G | | EN | | White |
| Guy | M | 59 | | Bac +3 à +5 | | Coach personal development / finance expert | | 2 | | N. Mamère | | N | | G | | N | | White |
| Dimitri | M | 48 | | Bac +3 à +5 | | Principal private secretary (arrondissement mayor) | | 3 | | L. Jospin | | Y | | G | | NE | | White |
| **PAR Activists 2** | | | | | | | | | | | | | | | | | | |
| Norbert | M | 65 | | Bac +2 | | Journalist (retired) | | 10 | | J.-M. Le Pen | | N | | B | | NE | | White |
| Jules | M | 46 | | Brevet/BEPC | | Photographer | | 3 | | R. Hue | | N | | NGNB | | World | | White |
| Adrien | M | 32 | | Bac +3 à +5 | | Editor/temporary teacher | | 2 | | N. Mamère | | N | | G | | NE | | White |
| Bertrand | M | 47 | | Bac +3 à +5 | | Communications adviser | | 5 | | J.-M. Le Pen | | N | | B | | N | | White |
| Pascal | M | 41 | | Bac +3 à +5 | | Engineer (researcher) | | 2 | | R. Hue | | N | | NGNB | | NE | | White |
| Emmanuel | M | 29 | | Bac +3 à +5 | | NGO manager | | 8 | | J. Chirac | | O | | G | | NE | | White |
| **BXL Working Class 1** | | | | | | | | | | | | | | | | | | |
| Justine | F | 38 | | Humanités sup gén | | Accountant (unemployed) | | 10 | | NV | | DK | | G | | NE | | Africa |
| Rose | F | 27 | | In training (advertisement) | | Receptionist | | 5 | | Cdh | | DK | | G | | NE | | White |
| Sidi | M | 27 | | Humanités inf | | Working man | | 4 | | Ecolo | | DK | | G | | E | | Maghreb |
| Marco | M | 43 | | Humanités inf | | Temporary worker | | 6 | | NV | | DK | | NGNB | | NE | | White |
| Saïd | M | 24 | | Humanités sup gén | | Youth activity worker (unemployed) | | NA | | PS | | Y | | G | | N | | Maghreb |
| Ali | M | 32 | | Graduat | | Care worker | | 2 | | PS | | N | | G | | NE | | Maghreb |
| **BXL Working Class 2** | | | | | | | | | | | | | | | | | | |
| Christophe | M | 24 | | Humanités sup pro/techn. | | Working man (unemployed) | | 5 | | MR | | N | | G | | NE | | White |
| Farouk | M | 28 | | Humanités sup pro/techn. | | Security guard | | 4 | | PS | | N | | NGNB | | NE | | Maghreb |
| André | M | 35 | | Humanités sup gén | | Woodworker (black market) | | 5 | | NV | | N | | NGNB | | World | | Other European |
| Dona | F | 56 | | Humanités sup pro/techn. | | Caretaker | | 7 | | MR | | DK | | G | | NE | | Other European |
| Ming | F | 24 | | Graduat | | Waitress | | 3 | | PS | | N | | G | | EN | | Asia |
| **BXL White Collars 1** | | | | | | | | | | | | | | | | | | |
| Faissal | M | 27 | | Licences/Master | | Temp/unemployed graphic designer | | 5 | | Ecolo | | Y | | G | | EN | | Maghreb |
| David | M | 24 | | Humanités sup pro/techn. | | Sergeant | | 4 | | PS | | Y | | G | | EN | | White |
| Victor | M | 28 | | Humanités sup gén | | Office worker | | NA | | Other | | DK | | G | | N | | White |
| Fabien | M | 26 | | Licences/Master | | IT adviser | | 7 | | MR | | Y | | NGNB | | EN | | White |
| **BXL White Collars 2** | | | | | | | | | | | | | | | | | | |
| Michèle | F | 26 | | Humanités sup gén | | Temp food industry | | 7 | | MR | | NV | | G | | NE | | White |
| Jonathan | M | 29 | | Humanités sup gén | | Computer technician | | 6 | | DK | | Y | | G | | E | | White |
| Tina | F | 32 | | Humanités sup gén | | Sales assistant (unemployed) | | 5 | | PS/MR | | Y | | G | | World | | Maghreb |
| Maria | F | 40 | | Humanités sup gén | | Office worker (television) | | 3 | | NV | | Y | | G | | NE | | Other European |
| Pierre | M | 54 | | Humanités sup gén | | Foreman | | 3 | | Cdh | | N | | B | | N | | NA |
| **BXL Managers 1** | | | | | | | | | | | | | | | | | | |
| Alban | M | 28 | | Licences/Master | | Engineer nuclear industry | | 8 | | MR | | DK | | G | | N | | White |
| Roger | M | 59 | | NA | | Executive electronics | | 4 | | PS | | Y | | G | | EN | | White |
| Jean-François | M | 29 | | Licences/Master | | NGO manager | | 3 | | Ecolo | | Y | | G | | EN | | White |
| Claire | F | 51 | | Doctorat | | General practitioner | | 7 | | Cdh | | DK | | G | | EN | | White |
| Franck | M | 40 | | Humanités inf | | Restaurant and shop manager | | 7 | | Ecolo | | DK | | G | | NE | | White |
| Valérie | F | 45 | | Licences/Master | | Journalist and researcher | | 6 | | Cdh | | Y | | G | | NE | | White |
| **BXL Managers 2** | | | | | | | | | | | | | | | | | | |
| Bruno | M | 29 | | Sciences  politiques &  Master en  commu. | | HR executive in SME | | 5/6 | | PS | | N | | NA | | NA | | Belgian |
| Fabio | M | 26 | | Traduction &  DES en études  Européennes | | Translator (unemployed) | | 2 | | ECOLO/PTB | | N | | NA | | NA | | Other European |
| Judith | M | 44 | | Licence en  communication | | Office  Manager –  HR | | 4 | | ECOLO/PS | | N | | NA | | NA | | African |
| Gaston | F | 60 | | Humanités | | Self-employed | | 7 | | MR | | N | | NA | | NA | | Belgian |
| **BXL Activists 1** | | | | | | | | | | | | | | | | | | |
| Aurélien | M | 28 | | Licences/Master | | Parliamentary attaché | | 8 | | MR | | Y | | G | | NE | | White |
| Stéphane | M | 30 | | Licences/Master | | Parliamentary attaché | | 7 | | MR | | Y | | G | | NE | | Asia |
| Clément | M | 33 | | Candidatures | | General practitioner | | 6 | | Cdh | | Y | | G | | NE | | White |
| Maxime | M | 25 | | Licences/Master | | Teacher | | 2 | | Ecolo | | N | | G | | World | | Maghreb |
| Simon | M | 30 | | Licences/Master | | Relations officer (party) | | 2 | | PS | | N | | G | | NE | | White |
| Romain | M | 20 | | Humanités sup gén | | Student | | 1 | | PS | | Y | | G | | NE | | White |
| **BXL Activists 2** | | | | | | | | | | | | | | | | | | |
| Gérard | M | 26 | | Licences/Master | | Shopkeeper | | 5 | | MR | | Y | | G | | NE | | White |
| Brandon | M | 27 | | Licences/Master | | Musician | | 2 | | PS | | N | | G | | EN | | Other European |
| Ludovic | M | 25 | | Graduat | | Secretary | | NA | | PTB-UA | | N | | G | | World | | NA |
| Vinciane | F | 29 | | Doctorat | | Elected representative | | 3 | | Ecolo | | N | | G | | Other | | Asia |
| Charles-Henri | M | 26 | | Licences/Master | | Legal adviser | | 5 | | Cdh | | Y | | G | | NE | | White |

**2. Heidi Mercenier’s participants**

Source: Mercenier, Heidi. (2019). *« C’est compliqué ! » : L’Union européenne vue par des jeunes Bruxellois Contribution à l’étude des rapports des citoyens à la politique.* Doctoral thesis, Université Saint-Louis – Bruxelles.

| **ID** | **Sex** | **Age** | **Education** | **Nationality** | **Employment status** | **Left-Right** | **Political interest**  **(0-10)** | **EU belonging** |
| --- | --- | --- | --- | --- | --- | --- | --- | --- |
| FG Anderlecht | | | | | | | | |
| Alexandre | M | 20 | ISCED 3 | Others | Student | 5 | 5 | Good thing |
| Amina | F | 19 | ISCED 3 | Belgian | Student | NA | 0 | Bad thing |
| Bilal | M | 26 | ISCED 5 | Belgian | Student | 5 | 7 | Good thing |
| Ilias | M | 18 | ISCED 3 | Belgian | Student | 4 | 7 | Neither a good nor a bad thing |
| Nabilla | F | 19 | ISCED 3 | Others | Student | NA | 4 | Good thing |
| Nisrine | F | 24 | ISCED 5 | Belgian | Student | 2 | 6 | Neither a good nor a bad thing |
| Waleed | M | 17 | ISCED 3 | Belgian | Student | 5 | 9 | Good thing |
| FG Ixelles | | | | | | | | |
| Aicha | F | 25 | ISCED 6 | Belgian | Student & part time employed | 5 | 6 | Good thing |
| Danielle | F | 17 | ISCED 3 | Belgian & other | Student | 3 | 4 | Good thing |
| Inaya | F | 19 | ISCED 5 | Belgian & other | Student | 5 | 4 | Neither a good nor a bad thing |
| Isabella | F | 22 | ISCED 5 | Belgian | Student | 4 | 6 | Neither a good nor a bad thing |
| Louis | M | 17 | ISCED 3 | Belgian | Student | 1 | 10 | Bad thing |
| Maël | M | 25 | ISCED 3 | Belgian | Employed part time | 3 | 2 | Good thing |
| Mun | M | 20 | ISCED 5 | Belgian | Student & part time employed | 3 | 7 | Good thing |
| FG Jette | | | | | | | | |
| Adil | M | 21 | ISCED 5 | Belgian | Student & part time employed | 4 | 7 | Bad thing |
| Catherine | F | 23 | ISCED 3 | Belgian | Student & part time employed | 7 | 5 | Good thing |
| Gabriel | M | 21 | ISCED 5 | Belgian | Student | NA | 5 | Good thing |
| Lucie | F | 17 | ISCED 3 | Belgian | Student | 3 | 7 | Good thing |
| Nathan | M | 26 | ISCED 5 | Belgian | Inactivity | 3 | 10 | Good thing |
| Nour | F | 19 | ISCED 5 | Belgian | Student & part time employed | 7 | 0 | Good thing |
| Yusef | M | 23 | ISCED 5 | Belgian | Student & part time employed | NA | 4 | Good thing |
| FG Molenbeek | | | | | | | | |
| Asma | F | 17 | ISCED 3 | Belgian | Student | 5 | 6 | Neither a good nor a bad thing |
| Mariam | F | 24 | ISCED 5 | Belgian | Employed part time | 3 | 9 | Good thing |
| Odomar | M | 17 | ISCED 2 | Belgian | Student | 4 | 0 | Good thing |
| Yassine | M | 16 | ISCED 3 | Belgian | Employed part time & student | 5 | 10 | Good thing |
| FG Saint-Josse | | | | | | | | |
| Abdel | M | 17 | ISCED 3 | Belgian | Student & employed | 4 | 4 | Good thing |
| Jordan | M | 22 | ISCED 2 | Belgian | Employed | 7 | 0 | Bad thing |
| Lila | F | 23 | ISCED 5 | Belgian | Student & employed | 7 | 7 | Good thing |
| Naima | F | 24 | ISCED 5 | Belgian & other | Unemployed | 4 | 3 | Good thing |
| FG Uccle | | | | | | | | |
| Elise | F | 17 | ISCED 3 | Belgian | Student & part time employed | 1 | 4 | Good thing |
| Julie | F | 24 | ISCED 5 | Belgian | Student & part time employed | 5 | 7 | Neither a good nor a bad thing |
| Nicolas | M | 21 | ISCED 5 | Belgian | Student | 7 | 7 | Good thing |
| Sophie | F | 22 | ISCED 5 | Belgian | Employed | 4 | 3 | Neither a good nor a bad thing |
| Théo | M | 19 | ISCED 5 | Belgian | Student & part time employed | 1 | 8 | Good thing |

**3. RESTEP’s participants**

Source: Beaudonnet, L., Belot, C., Caune, H., Dupuy, C., Houde, A., Le Corre Juratic, M .. & Van Ingelgom, V. (2022). Studying (De-)Politicization of the EU from a Citizens Point of View: A New Comparative Focus Group Study. *Politique européenne*, 75, 100-122.

| **Name** | **Age range** | **Gender** | **Education** | **Average Left-right score** | **Attitudes EU** |
| --- | --- | --- | --- | --- | --- |
| LLN_P_STU | 20 to 24 | 4 men, 5 women | Students | 2 left, 3 centre, 4 right | 9 pro-EU |
| LLN_SEQ_1, LLN_SEQ_2, LLN_SEQ_3 | 59 to 82 | 3 men, 4 women | High | 6 centre, 1 right | 6 pro-EU, 1 don’t know |
| LLN_WC | 25 to 36 | 4 men, 3 women | High | 3 left, 4 centre | 7 pro-EU |
| LLN_YU | 23 to 30 | 3 men, 3 women | High | 5 centre, 1 don’t know | 5 pro-EU |
| LLN_YP | 19 to 26 | 5 men, 1 woman | No diploma or professional | 1 left, 3 centre, 1 right | 4 pro-EU, 2 don’t know |
| GRE_P_STU | 18 to 26 | 5 men, 3 women | Students | 6 left, 1 centre, 1 no answer | 4 pro-EU, 2 against, 1 not good nor bad, 1 don’t know, |
| GRE_SEQ_1, GRE_SEQ_2, GRE_SEQ_3 | 61 to 77 | 4 men, 4 women | High | 2 left, 3 centre, 1 right, 1 don’t know | 5 pro-EU, 2 not good nor bad, 1 it depends, |
| GRE_WC | 28 to 33 | 3 men, 4 women | High | 1 left, 6 centre | 7 pro-EU |
| GRE_YU | 24 to 29 | 2 men, 3 women | High | 3 left, 2 centre | 5 pro-EU |
| GRE_YP | 22 to 36 | 3 men, 3 women | No diploma or professional | 1 left, 4 centre, 1 don’t know | 2 pro-EU, 1 against, 2 not good nor bad, 1 don’t know |
